# Study on the multitarget mechanism of alliin activating autophagy based on network pharmacology and molecular docking

**DOI:** 10.1111/jcmm.17573

**Published:** 2022-10-21

**Authors:** Bijun Cheng, Tianjiao Li, Fenglin Li

**Affiliations:** ^1^ Jilin Agricultural Science and Technology University Jilin China

**Keywords:** AKT, alliin, autophagy, molecular docking, network pharmacology

## Abstract

Due to the rapid development of bioinformatics, network pharmacology and molecular docking approaches have been successfully applied in the investigation of mechanisms of action. Here, we combined network pharmacology and molecular docking to predict the targets and reveal the molecular mechanism responsible for regulating autophagy by alliin. Based on the influence of alliin on autophagy, the targets of alliin were screened on the basis of different rules such as structural similarity by Pharmmapper, and genes associated with autophagy were collected from the GeneCards database. We focused on clarifying the biological processes and signalling pathways related to autophagy. Through the cytoHubba plug‐in and a series of integrated bioinformatics analyses, the top nine hub nodes with higher degrees were obtained. And finally, through the LibDock included in Discovery Studio 2019, molecular docking method was adopted to declare the reliability of the interaction between alliin and hub targets. The results suggest that alliin‐activated autophagy was possibly associated with pathways in cancer and the PI3K‐AKT signalling pathway. Furthermore, the potential targets (AKT1, MAPK14, MAPK, HSPA8, EGFR, HSP90AA1, SRC HSPA1A and HSP90AB1) were swimmingly screened on the basis of this practical strategy. Molecular docking analysis indicates that alliin can bind with AKT1 and EGFR with good binding scores. This network pharmacology could be an invaluable strategy for the investigation of action mechanisms of alliin‐activated autophagy. This study not only provides new and systematic insights into the underlying mechanism of alliin on autophagy, but also provides novel ideas for network approaches for autophagy‐related research.

## 1 INTRODUCTION

Autophagy is a precisely controlled homeostatic process in charge of the evacuation of damaged cytoplasmic constituents via the lysosomal pathway.^1^ This process has been a contributing factor in the regulation of various physiological roles containing cell growth, cytodifferentiation and cell viability. Cells could avoid stimuli in normal autophagy, but in the event of unrestrained autophagy or deficient autophagy, various human diseases may occur. Autophagy is thought to protect us against numerous kinds of human pathologies, including cancer, autoimmune disease, neurodegenerative disease and liver injury. Under certain conditions, take neurodegenerative disease for example, obviously, autophagy contributes to the dislodge of aggregate‐prone proteins that result in Huntington's and Parkinson's disease.^2^, ^3^ Nevertheless, autophagy plays a more complex role in cancer. The evidence suggests that autophagy may cause cancer in some cases, while in other cases, it might help tumour suppressor.^1^, ^4^, ^5^ In several acute hepatic injury animal model, autophagy helps to avoid hepatic acute damage.^6^, ^7^, ^8^ According to research, autophagy blocking significantly intensified APAP‐induced liver injury.^9^ Thus, protective autophagy exerts beneficial effects on pathological stresses and functions as a vital quality regulator in human cells.

Garlic (*Allium sativum* L.) is an aromatic herbaceous plant that is used all over the world as food and traditional medicine to treat numerous diseases. Alliin (s‐allyl‐l‐cysteine sulfoxide) is the highest sulphur‐containing, non‐protein amino acid, accounting for about 90% of the sulphur‐containing compounds in garlic. Alliin is considered to have a variety of biological properties including hypoglycaemic,^10^ lipid‐lowering,^11^ antibacterial,^12^ anti‐inflammatory,^13^ antioxidant,^14^ antitumor^15^ and immunomodulatory^16^ activities. Our previous work also found that alliin could improve the lipid metabolism disorders in HepG2 cells through the activation of the AMP‐activated protein kinase‐dependent pathway. Generous studies have confirmed the close relationship between autophagy and lipid metabolism.^17^, ^18^ However, the effect and mechanism of alliin on autophagy have not been clarified.

Autophagy can be indicated by changes of LC3 protein, which is needed for autophagosome formation. LC3II protein levels can indicate autophagosome number and detecting the conversion of LC3I to LC3II can suggest autophagic activity. Several specific substrates are gradually degraded by autophagy and the well‐characterized receptor is P62 (SQSTM1), which is a key indicator of autophagic flux. P62 selectively merges into autophagosomes by directly binding to the LC3 on autophagic membranes for subsequent degradation in autolysosomes.^19^ With the fast growth of bioinformatics, network pharmacology and molecular docking approaches have been successfully applied in the investigation of mechanisms of action. In order to uncover the effect and action mechanism of alliin on autophagy in HepG2 cells, we combined network pharmacology and molecular docking to predict the targets of alliin activated autophagy and reveal the molecular mechanism responsible for regulating autophagy by alliin.

## 2 MATERIALS AND METHODS

### 2.1 Reagents and antibodies

Alliin (Sigma‐Aldrich; product no. 1012950; purity >99.99%; molecular weight 177.22) was commercially ordered. LC3 (product no. 12741S), and P62 (product no. 16177S) were obtained from Cell Signalling Technology.

### 2.2 CCK8 assay

For the CCK8 assay, Cell Counting Kit 8 (Boster) was used according to the manufacturer's instructions. HepG2 cells were cultured in 96‐well plates at a density of 8 × 10^3^cells/well and then treated with different concentrations of alliin (0, 10, 25, 50, 100, 200, 400, 800 μM) for 22 h. The optical absorbance at 450 nm was measured after treatment of 10 μl CCK8 reagent for 2 h using a Tecan Infinite 200 Pro machine (TECAN).

### 2.3 Western Blot

Cells were seeded in 6‐well transparent plates. Then, cells were treated with alliin (25, 50, 100 μM) for 24 h. Western blot was performed as described previously.^20^ Briefly, cells were washed with PBS and were lysed in lysis buffer supplemented with PMSF. Proteins were separated by SDS‐PAGE and were electrically transferred to a PVDF membrane. The membranes were blocked for 1 h at room temperature in TBS containing 3% BSA and probed with specific primary antibodies (LC3 and P62) overnight at 4°C. Blots were then incubated with goat‐anti‐rabbit secondary antibodies. Bands were visualized using the ECL detection system (BLT Photon Technology).

### 2.4 Immunofluorescence staining

Cells were seeded in 24‐well transparent plates (Life Technologies; Thermo Fisher Scientific, Inc.) at a density of 5 × 10^3^ cells per well and were grown to achieve 80% confluency overnight. Then, cells were treated with alliin (25, 50, 100 μM) for 24 h, immunofluorescence staining was performed as previously described.^20^

### 2.5 Transmission electron microscope (TEM)

Cells were seeded in 6‐well transparent plates. Then, cells were treated with alliin (25, 50, 100 μM) for 24 h. Cells were fixed, dehydrated, embedded and double‐stained with uranyl acetate and lead citrate. At last, the autophagosomes were observed and photographed under H‐7650 TEM (Hitachi). Autophagosome is characterized by a double‐layer or multilayer vacuolar structure containing cytoplasmic components.

### 2.6 Data preparation

The chemical structure information of alliin (PubChem *CID*: 87310) was downloaded from NCBI PubChem database in sdf format.

### 2.7 Targets Predicted by PharmMapper and Genecards

The chemical structure information of alliin (PubChem *CID*: 87310) was submitted to the pharmmapper server (http://lilab‐ecust.cn/pharmmapper/submitjob.html). Then, the first 300 retained matching targets were selected for follow‐up study. Meanwhile, the targets related to autophagy were obtained by Genecards (http://www.genecards.org/) by inserting keyword “autophagy.” The list was then curated from duplicate gene entries of alliin‐related targets and autophagy‐related targets using the Funrich software version 3.1.3.

### 2.8 Gene and Pathway enrichment analysis

Gene enrichment was analysed by Metascape (metascape.org) with custom analysis. KEGG pathway enrichment was analysed by DAVID. Related pathways were obtained from KEGG pathway database (http://www.kegg.jp/, release 81.0) and Reactome (www.reactome.org).

### 2.9 Network construction and analysis

Metascape was used for Network construction. All networks were performed by Cytoscape and the MCODE algorithm. The cytoHubba plugin was adopted to rank targets in the PPI network by 12 means (EPC, MCC, DMNC, MNC, Closeness, Degree, BottleNeck, Betweenness, Stress, EcCentricity, Radiality and Clustering Coefficient), and the coincident genes were ultimately screened as the hub genes.

### 2.10 Sub‐Localization analysis of Hub targets by HPA

The Human Protein Atlas (HPA) (http://www.proteinatlas.org) is a complete database including a series of protein expression patterns of human proteins. The expression of each hub target was analysed by importing them into HPA.

### 2.11 Correlation exploring of hub targets and autophagy

The VarElect database was used to analyse gene list interpretation and scoring by entering search terms.^21^ We used the VarElect online tool to explore the relevance between hub targets and autophagy.

### 2.12 Molecular docking

Molecular docking was carried out according to the previous study.^22^ The molecular docking was performed with LibDock included in Discovery Studio 2019 (Accelrys). The LibDock is a flexible docking module. Smart Minimizer algorithm was used to minimize docked poses with CHARMm force field. The default parameter settings were used. The obtained docking poses were ranked by LibDock score.^22^ Further, to identify definite interacting residues of the receptor with bound ligand, a 2D diagram of docking was also performed.

## 3 RESULT

### 3.1 Effects of alliin on HepG2 cells viability

**FIGURE 1:**
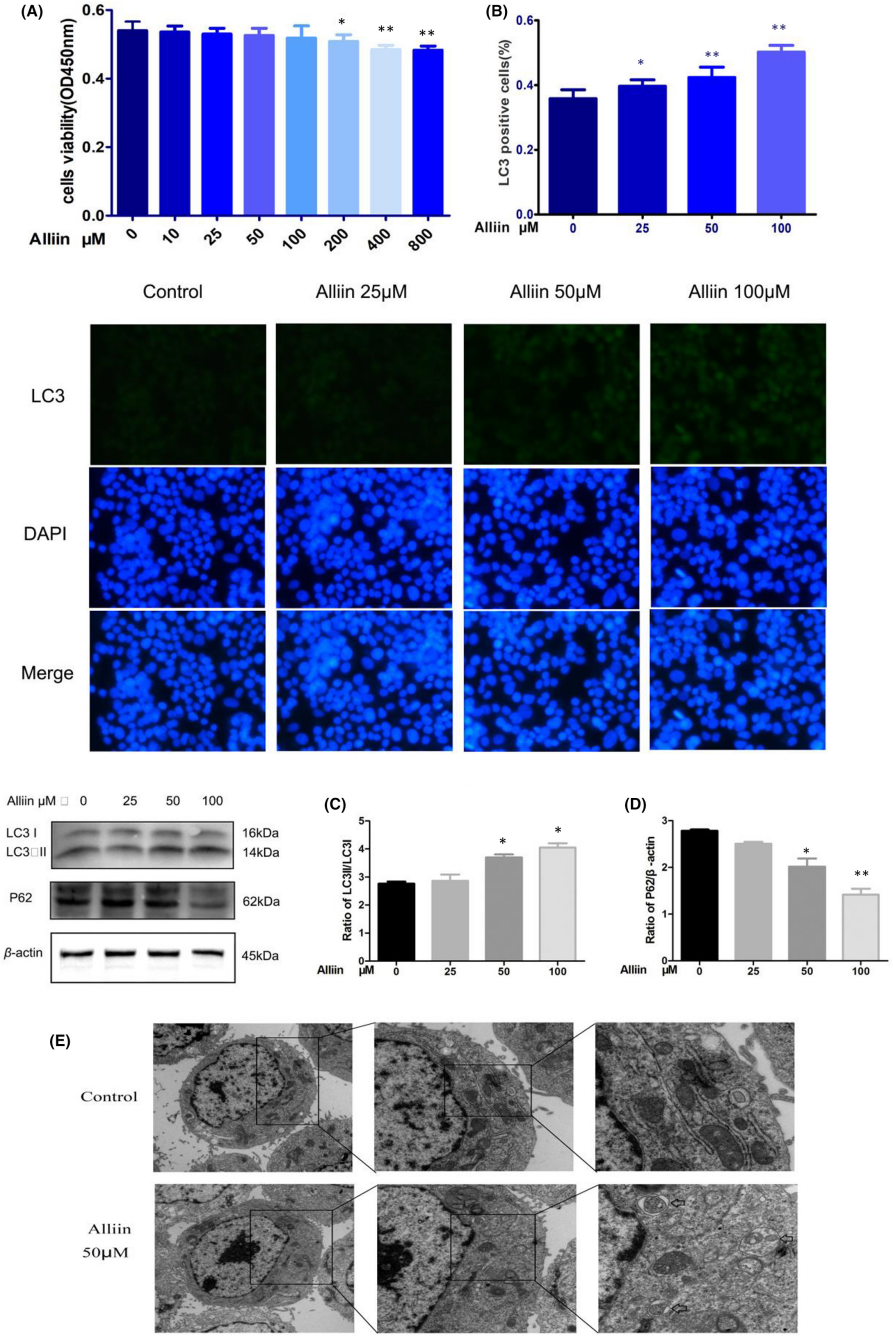
Alliin induced autophagy in HepG2 cells. (A) CCK8 assay for cellular viability; (B) Immunofluorescence staining results in cells; (C, D) Autophagy analysed by Western blotting analysis; (E) Images from transmission electron microscopy (TEM) images of characteristic autophagosomes (coarse arrows) in cells. N, nucleus. Mean ± SD, *n* = 3. *p < 0.05 vs. control, **p < 0.01 vs. control

The cytotoxic effect of alliin on HepG2 cells had been assessed byCCK8 assay. As shown in Figure 1A, alliin (0–100 μM) had no cytotoxic effect on HepG2 cells. However, treatment with alliin (200–800 μM) led to a marked decrease in the viability of HepG2 cells. Based on the results, we chose 25, 50 and 100 μM as the concentration range in the subsequent experiments.

### 3.2 Alliin‐induced autophagy in HepG2 cells

To explore whether alliin could induce autophagy in HepG2 cells, we calculated the ratio of LC3‐II/LC3‐I and expression of P62, a biochemical marker of autophagy, by Western blotting. Treatment with alliin at concentrations of 50 and 100 μM led to a significant increase of LC3‐II/LC3‐Iratio. Meanwhile, treatment with alliin diminished protein expression of P62 (Figure 1C,D).

To further analyse the effect of alliin on autophagy, we performed an immunofluorescence staining assay to analyse the distribution patterns of LC3. The addition of alliin significantly enhanced the level of LC3B positive dots. Quantitative comparison of LC3 positive cells in different treatment groups showed there is a significant improvement of the number of LC3B positive dots in alliin‐treated cells comparing with the control (Figure 1B).

Moreover, transmission electron microscopy (TEM), which is a standard method to measure autophagic process, disclosed more autophagosomes n alliin‐treated cells (Figure 1E). Autophagosomes were observed by TEM, which is a standard method to measure autophagic process. A higher magnification image obviously displayed the emergence of autophagosomes that encapsulating incompletely degraded cytoplasmic component. According to above, we come to the conclusion that alliin at concentrations of 50 and 100 μM induced autophagy in HepG2 cells.

### 3.3 Data preparation and construction

Firstly, with the help of Pharmmapper service, a total of 178 target proteins of alliin were received. Next, the GeneCard database was subsequently used to gain targets related to autophagy. After that, 100 targets associated with both autophagy and alliin were screened out and regarded as targets for network construction.

### 3.4 Enrichment analysis by GO, KEGG and Reactome

**FIGURE 2:**
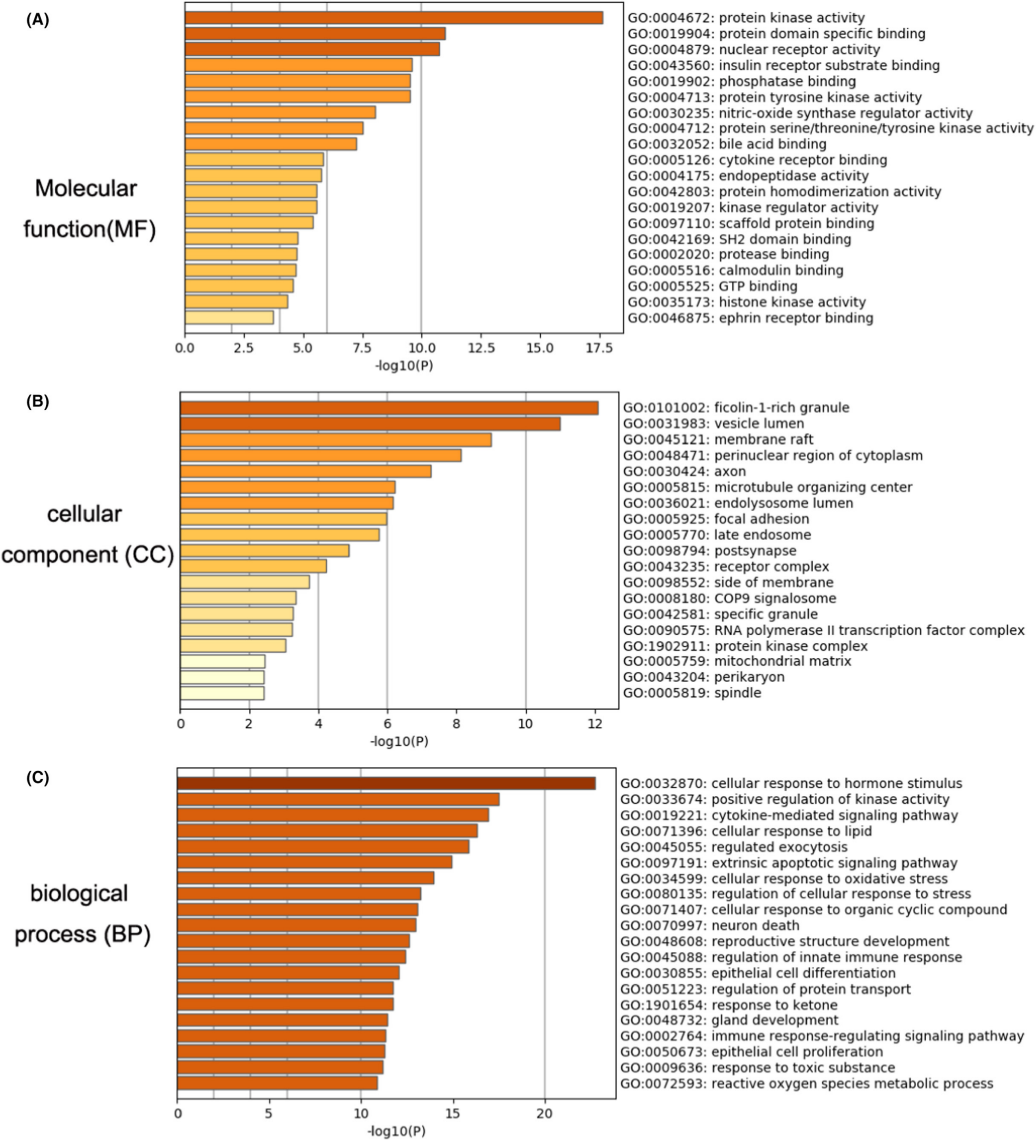
Top 20 Molecular function (MF), cellular component (CC) and biological process (BP) of 100 key targets. (A) Molecular function of 100 key targets; (B) Cellular component of 100 key targets; (C) cellular component of 100 key targets

**FIGURE 3:**
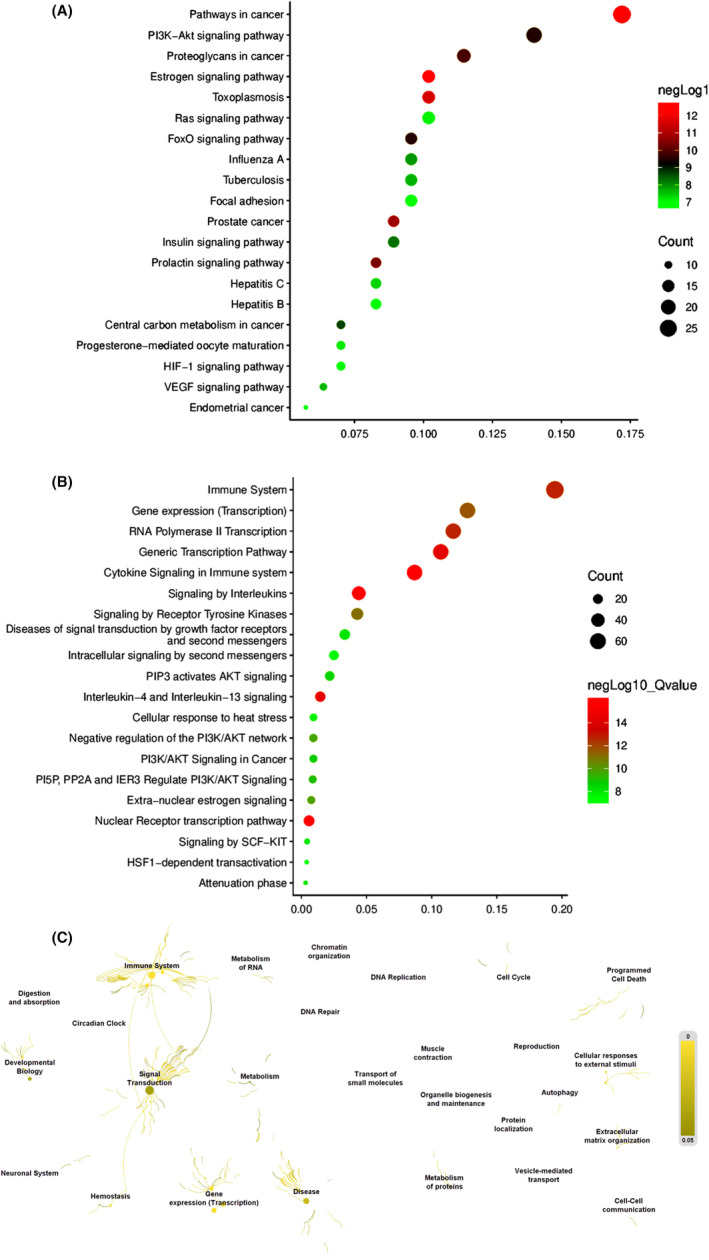
Pathway enrichment analysis of 100 targets. (A) Pathways enriched in the KEGG pathway; (B) Pathways enriched in Reactome pathway; (C) Terms ranked by *p* value in Reactome

The biological functions were also investigated by using Metascape, which contained molecular function (MF), cellular component (CC) and biological process (BP). For the MF, the principal differential expression proteins were enveloped in protein kinase activity (25%), protein domain‐specific binding (20%) and nuclear receptor activity (8%, Figure 2A). For the CC, most of proteins participated in processes, for instance, ficolin‐1‐rich granule (13%), vesicle lumen (15%), and membrane raft (13%, Figure 2B). For the BP, proteins were associated with cellular response to hormone stimulus (31%), positive regulation of kinase activity (25%) and cytokine‐mediated signalling pathway (27%, Figure 2C). Pathway enrichment analysis showed that pathways in cancer (17%), PI3K‐Akt signalling pathway (14%), proteoglycans in cancer (11%), oestrogen signalling pathway (10%) and toxoplasmosis (10%) were enriched in the KEGG pathway (Figure 3A). Immune system (19%), gene expression (transcription) (13%), generic transcription pathway (11%), RNA polymerase II transcription (12%) and cytokine signalling in immune system (9%) were gathered in the Reactome pathway (Figure 3B). In addition, to inquire thoroughly into the relationships between the terms, we screened a subset of enriched terms and drew a network plot, where terms with a similarity >0.3 are linked by edges. In the present study, we ranked the terms by *p* value and chose the top terms from each of the 20 clusters, with the restrictive rule that there are no more than 15 terms per cluster and no more than 250 terms in all (Figure 3C). The above molecular functions and biological processes revolved around the development of autophagy.

### 3.5 Construction of PPI network and selection of modules

For each given targets list, *PPI* enrichment analysis has been applied by using metascape. Eighty four nodes and 443 edges were constructed. We employed MCODE algorithm to discern closely connected network nodes. The MCODE networks identified for individual gene lists have been clustered, and MCODE score > 4 and nodes > 5 were set as the cut‐off criteria. So, the top two sub‐networks in the PPI network were selected for the subsequent GO and pathway enrichment analysis. (Figures S1 and S2).

Then, based on the cytoHubba plug‐in through 12 kinds of methods, the top nine hub nodes (HSP90AA1, HSP90AB1, SRC, HSPA8, EGFR, HSPA1A, MAPK1, AKT1, and MAPK14) were obtained.

### 3.6 Sub‐Localization Expression Analysis of Hub Genes

Based on HPA, the experimental evidence of the sub‐localization of hub targets (HSP90AA1, HSP90AB1, SRC, HSPA8, EGFR, HSPA1A, MAPK1, AKT1 andMAPK14) was obtained. The sub‐localization of HSP90AA1 and HSP90AB1 in human cells certified that HSP90AA1 and HSP90AB1 protein resided in the Cytosol based on the evidence provided by A‐431, U‐2 OS, U‐251 MG and MCF7 cells. Moreover, the other hub genes scattered at various zones of the cells, such as plasma membrane, nucleoplasm and nuclear speckles (Table 1).

**TABLE 1:** Distribution of hub genes in cells

Gene name	Cell lines	Main location
HSP90AA1	A‐431, U‐2 OS, U‐251 MG	Cytosol
HSP90AB1	MCF7, U‐2 OS, U‐251 MG	Cytosol
SRC	A‐431, A549, U‐2 OS	Cytosol, plasma membrane, cell junctions
HSPA8	CACO‐2，PC‐3， U‐2 OS	Vesicles，vesicles
EGFR	A‐431，U‐251 MG，U‐2 OS	Plasma membrane， cell junctions，
HSPA1A	A‐431, U‐2 OS, U‐251 MG	Nucleoplasm，cytosol
MAPK1	A‐431, U‐2 OS, U‐251 MG	Cytosol, plasma membrane, cell junctions
AKT1	A‐431, U‐2 OS, U‐251 MG	Nucleoplasm，microtubules
MAPK14	A‐431, U‐2 OS, U‐251 MG	Nuclear speckles，cytosol

### 3.7 Correlation analysis between Hub targets and Autophagy

**FIGURE 4:**
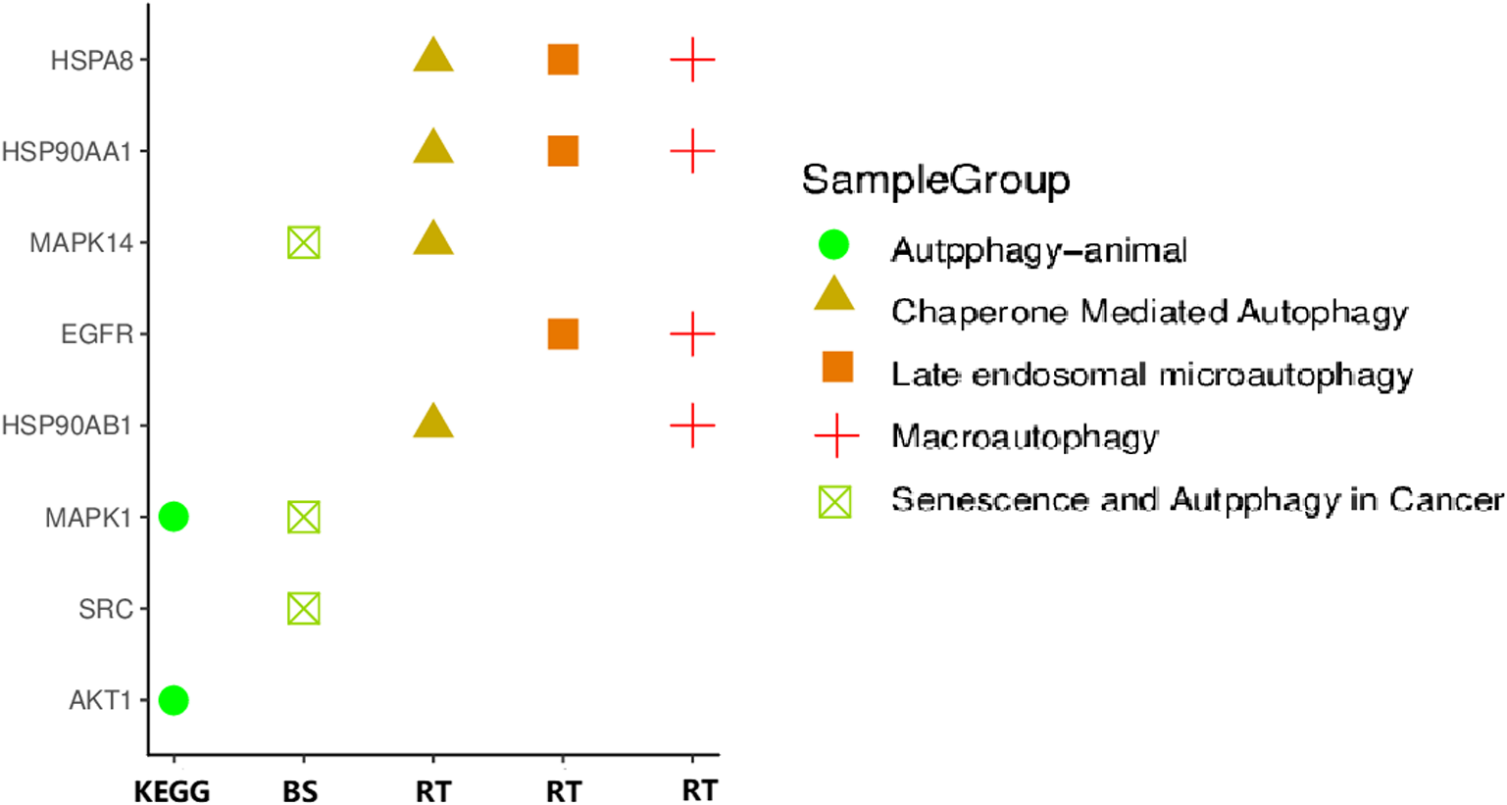
Correlation analysis between Hub targets and Autophagy by KEGG, Reactome (RT) and NCBI BioSystems (BS) Pathway analysis

Now nine hub genes were selected by cytoHubba plug‐in. To elucidate the direct correlation between hub targets and autophagy, Reactome, KEGG and NCBI BioSystems database were employed to display the distribution of hub genes in autophagy‐related pathways. Based on Reactome pathway analysis, it showed that HSP90AA1, HSP90AB1, HSPA8 and MAPK14 were involved in Chaperone Mediated Autophagy. HSP90AA1, HSPA8 and EGFR were gathered in late endosomal microautophagy. EGFR, HSP90AA1, HSP90AB1 and HSPA8 belonged to macroautophagy. Based on KEGG pathway analysis, it was detected that MAPK1 and AKT1 were involved in autophagy‐animal. Based on NCBI BioSystems pathway analysis, the results showed that MAPK14, SRC and MAPK1 belonged to senescence and autophagy in cancer (Figure 4).

In addition, the scores of these nine hub targets were also evaluated by using VarElect. This score was an indication of the strength of the connection between the targets and autophagy. As shown in Table 2, scores of AKT1, MAPK14, MAPK, HSPA8, EGFR, HSP90AA1 and SRC were >6. Despite lower HSPA1A and HSP90AB1 scores, there were also some literatures which confirmed their association with autophagy.^19^, ^23^, ^24^, ^25^, ^26^

**TABLE 2:** Scores of nine hub targets evaluated by VarElect

Symbol	Description	‐LOG10(*p*)	Score
AKT1	AKT serine/threonine kinase 1	2.66	13.17
MAPK14	Mitogen‐activated protein kinase 14	2.48	10.55
MAPK1	Mitogen‐activated protein kinase 1	2.41	9.62
HSPA8	Heat shock protein family A (Hsp70) member 8	2.30	8.17
EGFR	Epidermal growth factor receptor	2.30	8.16
HSP90AA1	Heat shock protein 90 alpha family class A member 1	2.16	6.90
SRC	SRC proto‐oncogene, non‐receptor tyrosine kinase	2.11	6.22
HSPA1A	Heat shock protein family A (Hsp70) member 1A	1.62	2.84
HSP90AB1	Heat shock protein 90 alpha family class B member 1	1.08	1.33

### 3.8 Molecular docking analysis

**FIGURE 5:**
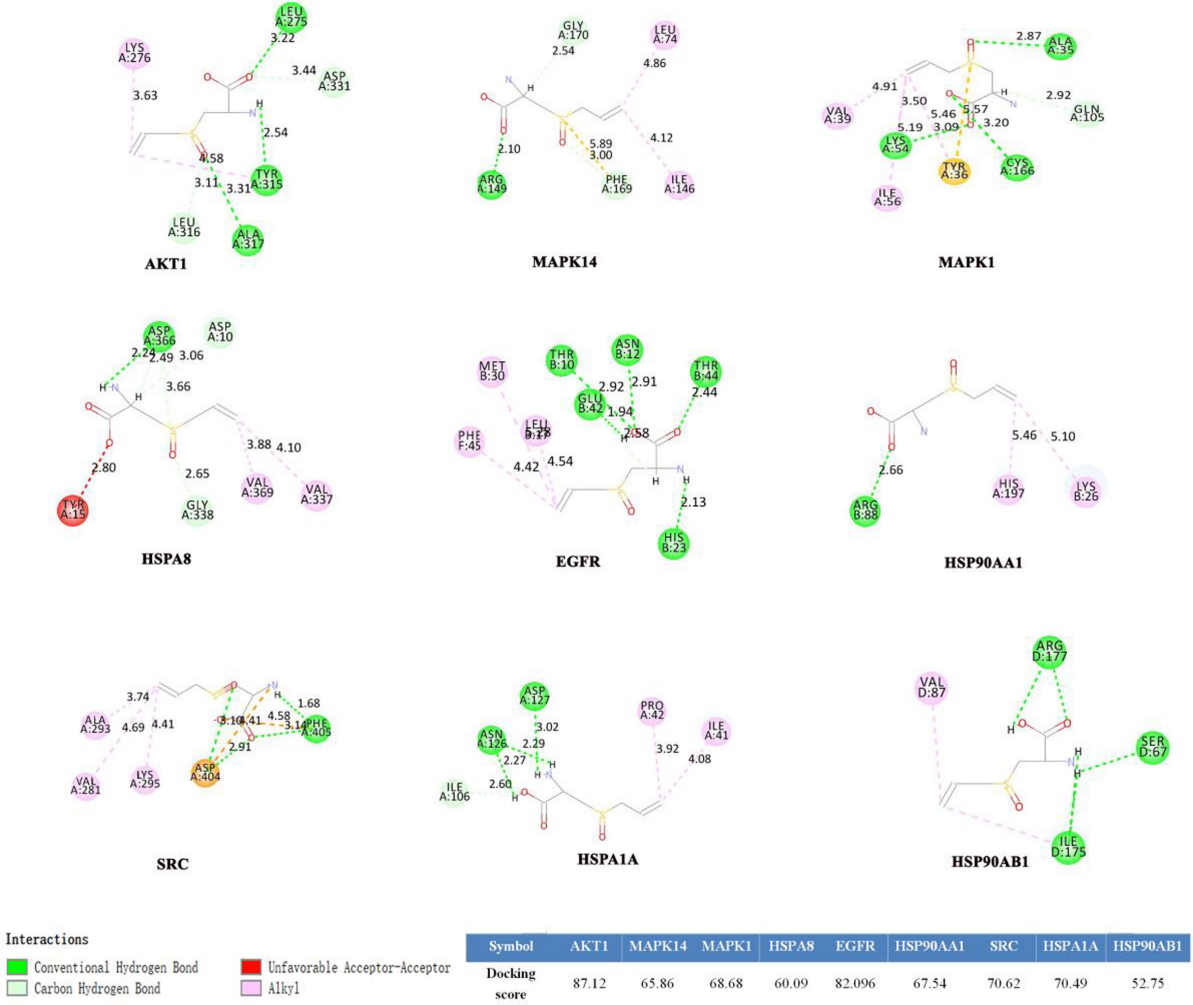
Molecular docking analysis of alliin and nine hub targets

We simulated the docking of alliin and nine hub targets to investigate the reliability of alliin‐target interactions. The molecular docking results revealed a high affinity of alliin towards a few proteins, especially AKT1, EGFR, SRC, MAPK1, and HSP90AB1. In the AKT1 protein binding site, alliin was found to be interacting with the Leu 275, Tyr 315 and Ala 317 residues of AKT1 with four H bonds. In the EGFR protein binding site, alliin was found to be interacting with EGFR through Thr 10, Glu 42, Asn 12, Thr44 and His 23. In the SRC protein binding site, alliin was found to be interacting with SRC through Phe 405. The hydrogen bonding between alliin and MAPK1 indicates a stable interaction with a total of three bonds, involving Ala 35, Lys 54 and Cys 166. On the contrary, alliin‐HSP90AB1 complex showed a binding affinity with three residues including Arg 177, SER 67 and Ile 175 via hydrogen bonds. And the binding modes of other hub targets and alliin were displayed in Figure 5. With the interaction of alliin, AKT1 and EGFR showed higher LibDock score, indicating stronger binding forces.

## 4 DISCUSSION

With the increasing studies on autophagy, it is a firm belief that impairment and dysregulation of autophagy pathways are highly relevant for many human diseases, such as cancer, lysosomal storage diseases, neurodegenerative diseases and significantly associated with processes such as aging, development and immunity. The autophagy formation process requires many autophagy‐related genes. In this study, by immunofluorescence staining and TEM, we found that alliin can activate autophagy in HepG2 cells. Then, we applied numerous bioinformatics and molecular docking methods to explore the mechanism of alliin on autophagy, especially its key targets. Although this study only made a thorough inquiry of the mechanism of alliin on autophagy from the point of molecular informatics, in our opinion, this study still deserves attention in a few hints.

Firstly, based on the influence of alliin on autophagy, the targets of alliin were selected depending on different rules such as structural similarity by Pharmmapper, and genes associated with autophagy were collected from GeneCards database. The 100 intersection genes of alliin and autophagy may be the potential targets of alliin activated autophagy. Then, the 100 genes were analysed by Metascape, DAVID and Reactome. In order to investigate the action mechanism of alliin, we concentrated on analysing the biological processes and signalling pathways related to autophagy. Based on the reactome pathways analysis, the highly enriched pathways of alliin were related to Immune System, Gene expression (Transcription), Generic Transcription Pathway, RNA Polymerase II Transcription and Cytokine Signalling in Immune system. From KEGG pathway enrichment, the dramatically enriched pathways of alliin were related to pathways in cancer, oestrogen signalling pathway, PI3K‐Akt signalling pathway, Toxoplasmosis and Proteoglycans in cancer. Through PPI network construction and MCODE scores, which represent importance, module 1 and module 2 were found to play major roles in the PPI. GO and pathway analyses were also performed in module 1 and module 2. KEGG signalling pathway analysis of module 1 also revealed the importance of pathways in cancer and PI3K‐Akt signalling pathway. It is found that autophagy can suppress or accelerate cancer cell proliferation or tumorigenesis, and the function of autophagy in cancer depends on the environment.^27^ Therefore, researchers suggest that autophagy regulation may be a new treatment strategy for some malignant tumours.^28^, ^29^ Previous studies have reported that the activation of autophagy is related to suppression of PI3K/AKT/mTOR signalling pathway in rats with OA.^30^ Li et al.^31^ found that ligustrazine activated autophagy by regulating the PI3K/AKT/mTOR signalling pathway. Similarly, allicin, which is formed from alliin by alliinase, could trigger autophagy‐dependent cell death through suppressing the Akt/mTOR signalling pathway.^32^ Taken together, our study provided theoretical evidence that the effect of alliin activating autophagy was possibly associated with pathways in cancer and the PI3K‐Akt signalling pathway.

Through the cytoHubba plug‐in and a series of integrated bioinformatics analyses, the top nine hub nodes with higher degrees were obtained. The VarElect database was adopted to examine the correlation between nine hub targets and autophagy. Among the nine hub targets, the scores of AKT1, MAPK14, MAPK, HSPA8, EGFR, HSP90AA1 and SRC > 6, showing a high correlation with autophagy.

AKT1 located on chromosome 19. GO Biological Function of AKT1 was negative regulation of autophagy. Castino R et al.^33^ showed that Akt was violently phosphorylated in the first half hour upon exposure to H_2_O_2_, at the same time, accompanying by autophagy inhibition. Huang Y.et al.^34^ found that modulation of autophagy is possibly regulated by interruption of the Akt/mTOR pathway. Wang KF. et al.^35^ provided evidence that XC‐302 can activate autophagy in CNE‐2 by inhibiting the PI3K/AKT/mTOR signalling pathway.

MAPK1 located on chromosome 22, and MAPK14 located on chromosome 06. MAPK1 and MAPK14 belonged to MAPK pathway. MAPK14 was a serine/threonine kinase which inhibited the lysosomal degradation pathway of autophagy. Jose Félix Moruno‐Manchón et al.^36^ determined the connection between the MAPK pathway and the mediation of autophagy through mAtg9 and p38IP. Yi Luo et al.^37^ revealed that the induction of autophagy depends on the activation of MAPK14 pathway. Yingli He et al.^38^ clarified that p38α MAPK activity is necessary for LPS‐induced morphological changes and the production of IL‐1β by primary microglia in vivo and in vitro, which is associated with the p38α MAPK‐dependent suppression of autophagy. The effect of MAPK1 on autophagy was studied very early. Hiroshi Aoki et al.^39^ found that interruption of the MAPK1/MAPK3 pathway suppressed curcumin‐induced autophagy and led to apoptosis. To Sing Fung, the anti‐apoptotic extracellular signal‐regulated MAPK1 also contributed to infectious bronchitis virus‐induced autophagy.^40^ Furthermore, Saray et al.^41^ found that alliin prevented LPS‐induced inflammation in 3 T3‐L1 adipocytes by decreasing phosphorylation of MAPK, which also provided a reference for the selection of MAPK as a potential target of alliin.

HSPA8 located on chromosome 11. It was protein targeting to lysosome involved in chaperone‐mediated autophagy. Interaction between FUNDC1 and chaperone HSPA8 accelerated the mitochondrial translocation of unfolded cytosolic proteins to form MAPAs, which were separated from mitochondria in the form of FIS1 dependence and then degraded by autophagy.^42^ Ina Oehme et al.^43^ Provide evidence that HDAC10 enhances autophagy‐induced survival in neuroblastoma cells through interaction with heat shock HSPA8.

EGFR located on chromosome 07. EGFR plays a key role in the transition between hypoxic autophagy‐induced cell survival and cell death.^44^ EGFR can ectopically activate downstream signalling oncogenic cascades such as PI3K/Akt/mTOR pathway, thereby participate in the autophagy‐mediated process.^45^ Wen Xu et al.^46^ showed that the interaction between EGFR and the autophagy inhibitor protein (rubicon) led to the release of beclin‐1, thereby activating autophagy

HSP90AA1 located on chromosome 14. And HSP90AB1 located on chromosome 06. Heat shock protein 90 (Hsp90) is a molecular chaperone that regulates the stability of signalling proteins. GO Biological Function of HSP90AA1 is chaperone‐mediated autophagy. Hsp90 has an evolutionarily conserved domain, through which it interacts with Beclin 1, to regulate the homeostasis of Beclin 1. Thus, Hsp90 plays a novel role in autophagy by maintaining the stability of Beclin 1.^47^

SRC located on chromosome 20.Src phosphorylates mATG9 at Tyr8 to control its endocytic and primary transport in unstressed conditions. In case of starvation, phosphorylation of mATG9 at Tyr8 by SRC and at Ser14 by ULK1 coordinated to promote combination of mATG9 and the AP1/2 complex, resulting in redistribution of mATG9 from the plasma membrane and juxta‐nuclear region to the peripheral pool for autophagy activation.^48^ In addition, endoplasmic reticulum stress leads to epithelial‐mesenchymal transition through activating autophagy.^49^

HSPA1A located on chromosome 06. Autophagy‐induced stress raised intracellular levels of acetylated inducible HSPA1A, which bound to the Beclin‐1‐Vps34 complex. Acetylated HSPA1A also mobilized E3 ligase to activate Lys840 SUMOylation and enhance Vps34 activity bound to Beclin 1. In another word, acetylated HSPA1A and KAP1‐induced Vps34 SUMOylation were essential for autophagosome formation in autophagy.^24^

To explore the mechanism of the interactions between alliin and hub targets, we adopted molecular docking method to confirm the dependability of the interaction. Also, the result indicates that alliin can bind to AKT1, and EGFR with good binding scores. The accurate mechanism in view of molecular docking also demands further validation in biological experiments.

## 5 CONCLUSION

In conclusion, the effect and underlying mechanism of alliin on autophagy in HepG2 cells were investigated in this study by combining experimental operation and network pharmacology prediction. Our study demonstrated alliin activated autophagy in HepG2 cells and the potential molecular mechanism was possibly related to pathways in cancer and PI3K‐Akt signalling pathway. Furthermore, the potential targets (AKT1, MAPK14, MAPK, HSPA8, EGFR, HSP90AA1, HSPA1A and HSP90AB1) were finally elected based on this practical strategy. Collectively, the current study provides a systematic and visual overview of potential targets and signalling pathways associated with the activities of alliin activating autophagy. Also, there are some limitations in our study. Based on the topological analysis and key targets analyses of the constructed network, we docked nine hub targets with alliin. In the future experiment, we should not only use molecular dynamics method to verify the binding ability of alliin with hub targets, but also investigate the mechanism with nine hub targets based on the results of further biological experiments.

## AUTHOR CONTRIBUTIONS

**Bijun Cheng:** Data curation (lead); writing – original draft (lead). **Tianjiao Li:** Funding acquisition (lead); writing – review and editing (lead). **Fenglin Li:** Investigation (lead); supervision (lead).

## CONFLICT OF INTEREST

The authors confirm that there are no conflicts of interest.

## Supporting information


Appendix S1

## Data Availability

The data that support the findings of this study are available from the corresponding author upon reasonable request.
