# Supplementary material for: Study on the multitarget mechanism of alliin activating autophagy based on network pharmacology and molecular docking
Source: J Cell Mol Med. 2022 Oct 21;26(22):5590–601. doi: 10.1111/jcmm.17573 (PMC9667524; doi:10.1111/jcmm.17573)
Supplement: Supplementary file 1 — Appendix S1 [file JCMM-26-5590-s001.docx]

### Study on the Multitarget Mechanism of Alliin activating autophagy Based on Network Pharmacology and Molecular Docking

**Supplementary text**

In MCODE1, for the MF, the targets anticipated in ATP binding(39%), nitric-oxide synthase regulator activity (11%), transmembrane receptor protein tyrosine kinase activity(11%), and steroid binding (7%). For the CC, the majority of proteins were involved in nucleus (21%), plasma membrane (18%), and cytosol (14%). For the BP, proteins were closely linked to positive regulation of transcription from RNA polymerase II promoter (17%), transmembrane receptor protein tyrosine kinase signaling pathway(14%), and positive regulation of nitric oxide biosynthetic process (11%)(Fig. S1A). Furthermore, the most significantly enriched pathways in MCODE1 were analyzed by KEGG and Reactome. Pathway enrichment analysis in Fig. S1 (B) showed that Signaling by Receptor Tyrosine Kinases(4%), diseases of signal transduction by growth factor receptors and second messengers(3%), and negative regulation of the PI3K/AKT network(1%) were enriched in the Reactome pathway. PI3K-Akt signaling pathway(46%); pathways in cancer(39%), and prostate cancer(35%) were gathered in the KEGG pathway.(Fig. S1B).

In MCODE2, for the MF, the targets anticipated in ATP binding(38%), transmembrane receptor protein tyrosine kinase activity (8%), and protein serine/threonine kinase activity (13%). For the CC, the majority of proteins were involved in perinuclear region of cytoplasm (17%), mitochondrion (17%), and cytosol (17%). For the BP , proteins were closely linked to protein autophosphorylation (13%), signal transduction in response to DNA damage(9%), and positive regulation of axon extension (9%) (Fig. S2A). Pathway enrichment analysis in Fig. S2(B) showed that intracellular signaling by second messengers(3%), PIP3 activates AKT signaling(2%), and negative regulation of the PI3K/AKT network(1%) were enriched in the Reactome pathway. Ras signaling pathway(26%), Prolactin signaling pathway(22%), and Insulin signaling pathway(22%) were gathered in the KEGG pathway.(Fig. S2B).


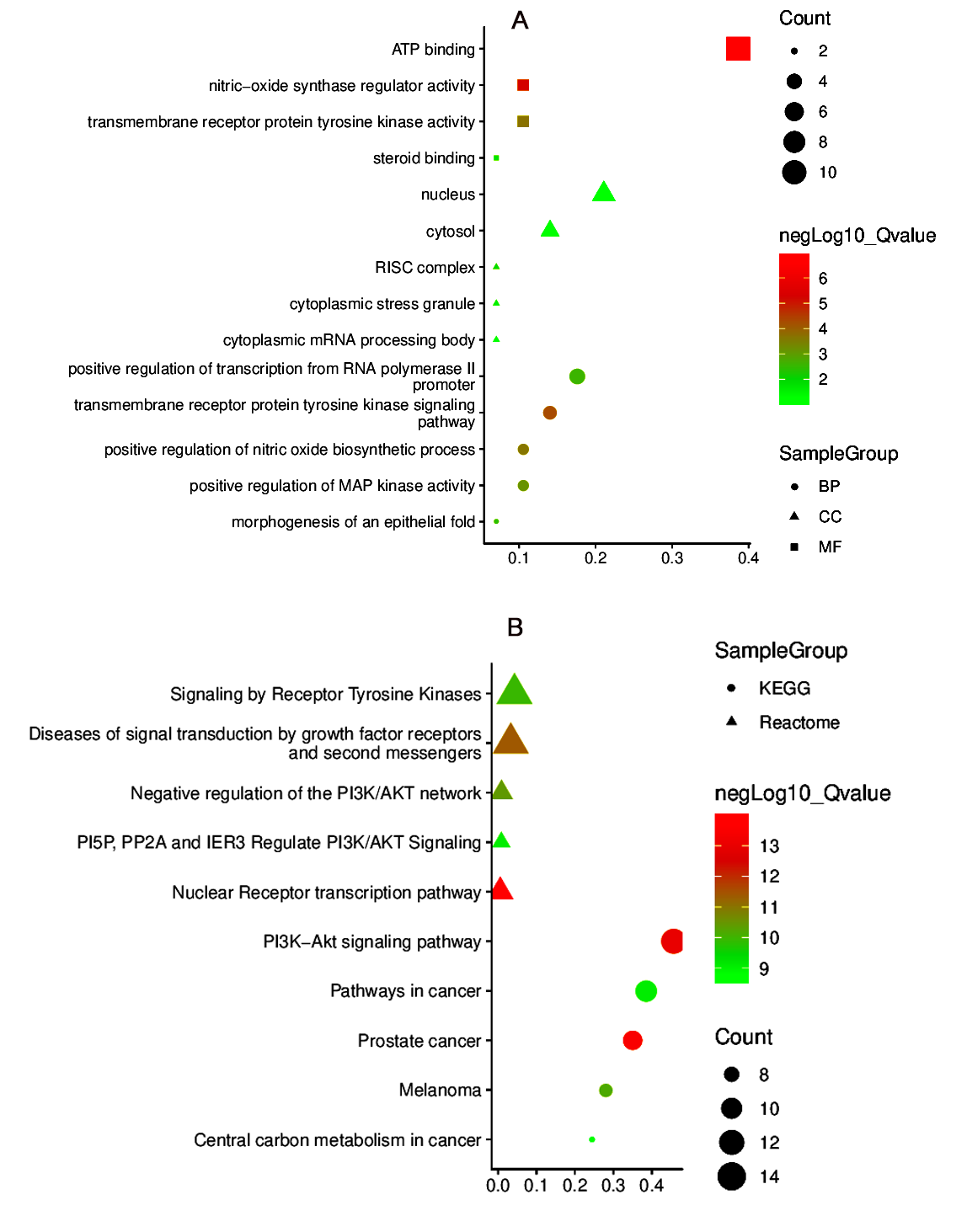


Fig. S1 Biological functions and Pathway enrichment analysis of MCODE1


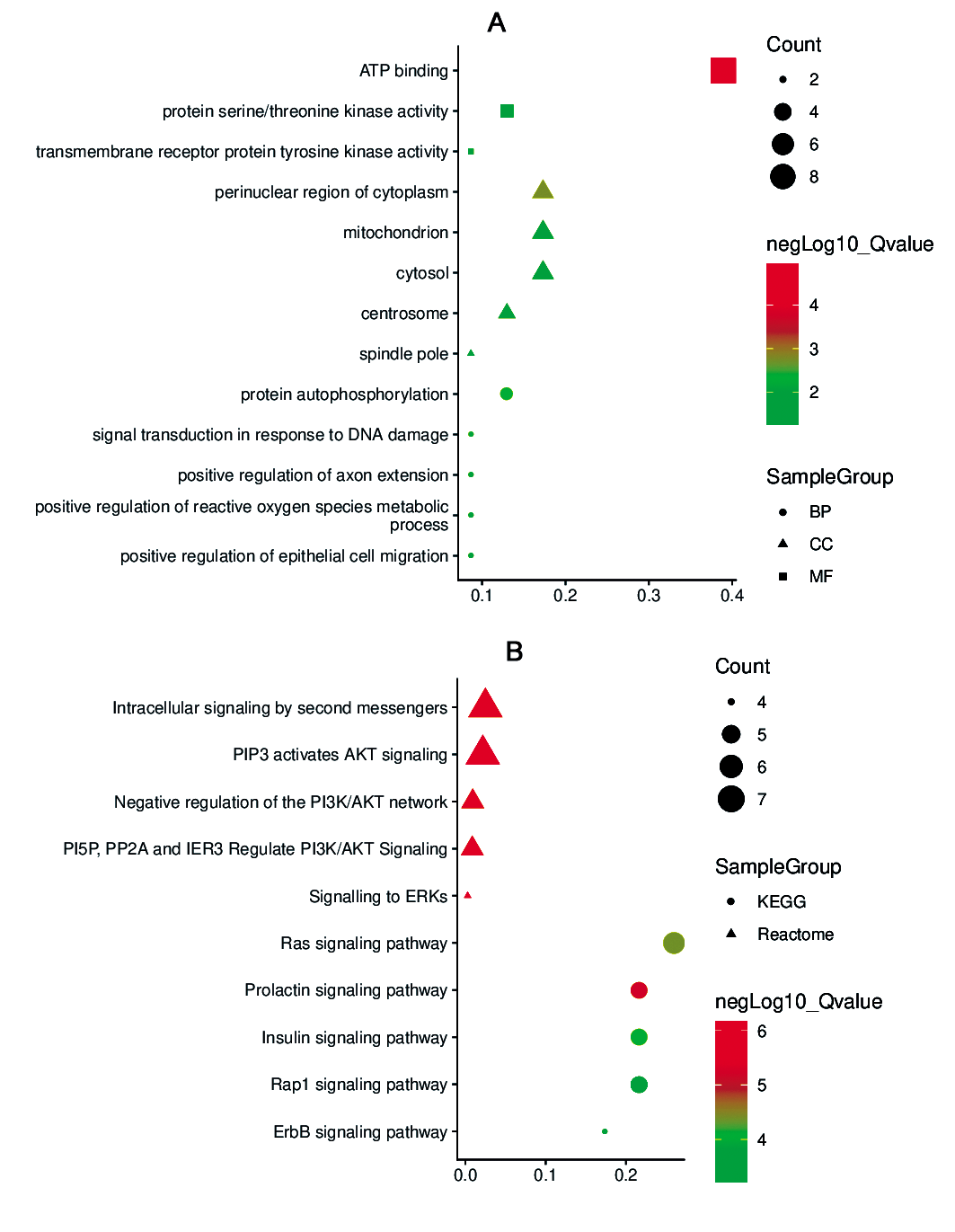


Fig. S2 Biological functions and Pathway enrichment analysis of MCODE2
